# CMV Colitis: A Rare Complication of Azacitidine and Venetoclax Chemotherapy

**DOI:** 10.3390/hematolrep14030029

**Published:** 2022-06-23

**Authors:** Mustafa Nissar Bankur, Archie Keeling, Khoodoruth Mohamed Adil Shah, Daniele Avenoso

**Affiliations:** 1 Department of Haematological Medicine, King’s College Hospital NHS Foundation Trust, London SE5 9RS, UK; mustafa.bankur@nhs.net; 2 Neurosciences Department, King’s College Hospital NHS Foundation Trust, London SE5 9RS, UK; 3 Radiology Department, King’s College Hospital NHS Foundation Trust, London SE5 9RS, UK; archie.keeling@nhs.net; 4 Department of Psychiatry, Hamad Medical Corporation, Doha 00000, Qatar; mkhoodoruth@hamad.qa

**Keywords:** AML, CMV, venetoclax

## Abstract

Herein, we present a case of cytomegalovirus (CMV) colitis that occurred after two cycles of azacitidine and venetoclax in a 64-year-old woman affected with acute myeloid leukaemia (AML) secondary to a previous diagnosis of a hypoplastic myelodysplastic syndrome (hypo-MDS). This patient never had detectable CMV viraemia, and there was no evidence of immune deficiency that could justify this opportunistic infection. Additionally, this is most likely the first report describing CMV colitis in a patient treated upfront with azacitidine and venetoclax.

## 1. Introduction

Cytomegalovirus (CMV) reactivation is a common infective complication following haematopoietic stem cell transplant (HSCT). In both autologous and allogeneic settings, the estimated incidence of it ranges between 12% and 37%, respectively [1].

CMV reactivation and disease in the non-transplant setting for malignant blood diseases is quite uncommon, with few data available. Risk factors for CMV reactivation/disease include advanced disease, poor performance status, and the use of high-dose steroids, fludarabine, alemtuzumab (antiCD52 monoclonal antibody), bortezomib (proteasome inhibitor), and rituximab (anti-CD20 monoclonal antibody) [2].

Azacitidine (a hypomethylating agent) and venetoclax (anti-BCL2 molecule) are not commonly associated with opportunistic infections including CMV [3]. Indeed, this combination is offered to elderly or unfit patients affected with high-risk myeloid neoplasms.

Here, we present a case of CMV colitis following treatment with azacitidine and venetoclax for acute myeloid leukaemia (AML). While reviewing the literature, we found only one prior case report mentioning the occurrence of CMV colitis following azacitidine single-agent administration [4]. However, it is important to highlight that CMV reactivation/disease is most likely an under-diagnosed condition in AML settings before HSCT, as previously reported [5]. Even if this is a rare complication, it is worth highlighting that CMV colitis arising post-chemotherapy has been reported previously, as shown in Table 1.

## 2. Case Presentation

A 64-year-old woman affected with AML secondary to a previous diagnosis of hypo-MDS presented to the emergency department with a recent onset of bloody diarrhoea, together with abdominal cramps, decreased appetite, and a 10 kg weight loss, after receiving two cycles of induction chemotherapy with azacitidine and venetoclax. Previously, she had two admissions for neutropenic sepsis after cycles one and two of chemotherapy, as a result of which she received piperacillin/tazobactam. When she was initially diagnosed with hypo-MDS in 2017, the patient was first treated with a ciclosporin single agent for nine months which was then stopped due to lack of response. After stopping ciclosporin (April 2019), she was on regular red cell transfusion due to the patient’s refusal of allogeneic stem cell transplantation. Eighteen months following the failure of ciclosporin, her disease progressed to AML, and therefore, she was started on azacitidine and venetoclax as induction chemotherapy.

At the onset of diarrhoea, her vital signs were stable, but she looked frail and dehydrated. She was not pale or jaundiced. Her respiratory and cardiovascular examinations were within normal limits. She had epigastric tenderness on abdominal examination without peritoneal signs.

Her initial laboratory investigations included haemoglobin 101 g/L, platelet 404 × 10^9/L, white blood cells 7.79 × 10^9/L (complete blood counts with differentials are provided in Table 2), and c-reactive protein (CRP) 53 mg/L. The renal functions, liver function tests, and thyroid function tests were all within normal limits.

Her stool was tested for invasive bacterial infections including Shigella, Salmonella, and *E. coli* 0157, which were all negative. It was also negative for C. difficile toxins. The test results were negative for faecal adenovirus DNA and norovirus RNA, and no cryptosporidium was found on Ziehl–Neelsen staining. Faecal calprotectin test was elevated at 2260 μg/g (normal < 50 μg/g). The full panel of the stool test is summarised in Table 3 below.

**Figure 1:**
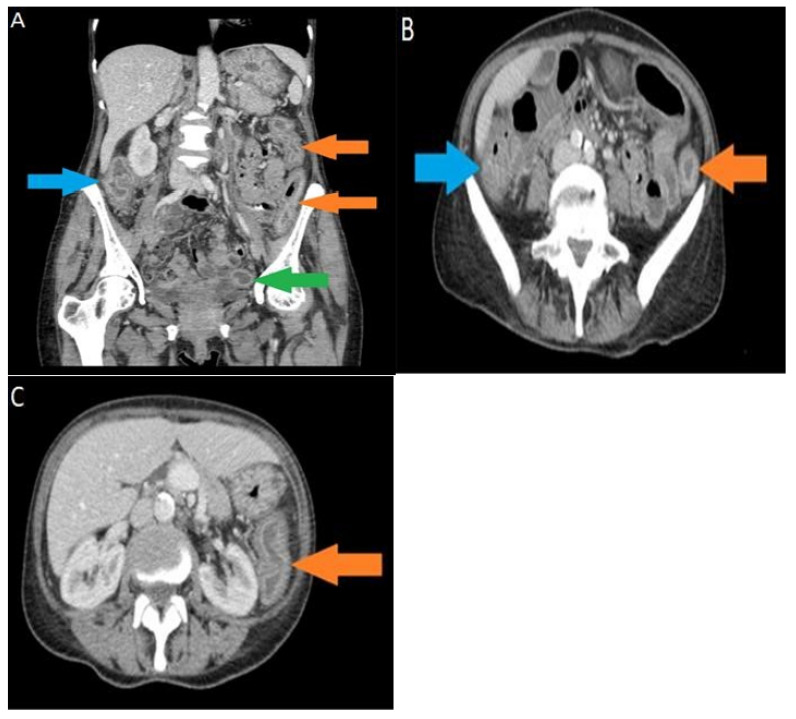
(**A**) Selected coronal CT image, acquired in the portal venous phase, shows marked bowel wall thickening and mucosal hyperenhancement in the ascending colon (blue arrow), descending colon (orange arrows), and sigmoid colon (green arrow), in agreement with the diagnosis of active multifocal colitis; (**B**) selected axial CT image, acquired in the portal venous phase, shows marked bowel wall thickening and mucosal hyperenhancement in the hepatic flexure of the large bowel (blue arrow) and splenic flexure of the large bowel (orange arrow), in agreement with the diagnosis of active multifocal colitis; (**C**) selected axial CT image, acquired in the portal venous phase, shows marked bowel wall thickening and mucosal hyperenhancement in the descending colon (orange arrow), in agreement with the diagnosis of active colitis.

**Figure 2:**
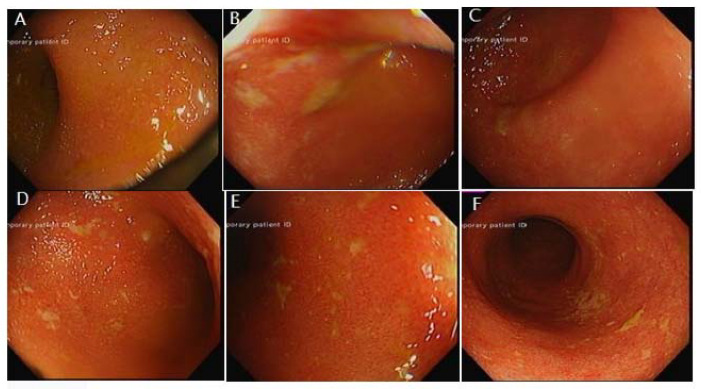
From mid-descending colon to rectum (**A**–**F**): There was a continuous superficial inflammation. Loss of vascular pattern occurred throughout. Aphthous ulcers were seen but only superficially.

A CT scan abdomen pelvis with contrast was performed, and the findings of diffusely marked bowel wall thickening and mucosal hyperenhancement were in agreement with a diagnosis of pancolitis (Figure 1). A sigmoidoscopy was carried out, leading to the findings of continuous superficial inflammation and superficial aphthous ulcers in the colon (Figure 2). Biopsies were taken in series to rule out CMV colitis.

CMV DNA level was also tested from the blood, which was found to be negative.

### 2.1. Differential Diagnosis

Given that the patient had a previous history of antibiotic use, C. difficile colitis was believed to be the most likely cause, but her stool was negative for C. difficile toxins. A multistep approach was used while testing for C. difficile as per our institution’s policy. This included enzyme immunoassay testing for both C. difficile glutamate dehydrogenase (GDH) and toxins A and B.

While GDH testing has a high sensitivity (100%), it cannot distinguish between toxigenic and non-toxigenic strains [16]. It is used together with toxin A and B enzyme immunoassay which has a high specificity (92% to 98%) [17]. The stool sample for our patient tested negative for both GDH and toxins A and B, thus confidently ruling out C. difficile infection.

Infective causes were ruled out with the stool tested negative for invasive bacterial and viral pathogens. Although the patient was not considered to be severely immunocompromised, a biopsy taken from the colon was still tested for CMV.

The high faecal calprotectin and findings of sigmoidoscopy pointed towards inflammatory bowel disease (IBD) as a possible cause.

### 2.2. Treatment, Outcome, and Follow Up

The patient’s symptoms did not improve on supportive therapy alone, and considering the findings suggestive of IBD (together with the absence of CMV in the peripheral blood), she was started on intravenous methylprednisolone 20 mg once daily (od) and oral budesonide 3 mg three times daily (tds). She had improvement in the frequency of her diarrhoea and a slight increase in dietary intake after a few days of steroid therapy.

One week later, despite the presence of anti-inflammatory therapy, she still reported bloody diarrhoea, although the frequency was decreased. Her biopsy was reported at that time with findings of diffuse active/chronic colitis with cryptitis and crypt abscess formation. Features of chronicity (such as crypt architectural distortion or a basal lymphoplasmacytosis) were not prominent. No granulomas were identified. Immunohistochemical stain for CMV was positive in scattered cells, and a number of herpes viral inclusions were noted in glandular epithelial cells. These findings were in agreement with a diagnosis of CMV colitis. She was started on intravenous ganciclovir for 14 days in total and steroid treatment was discontinued.

**Figure 3:**
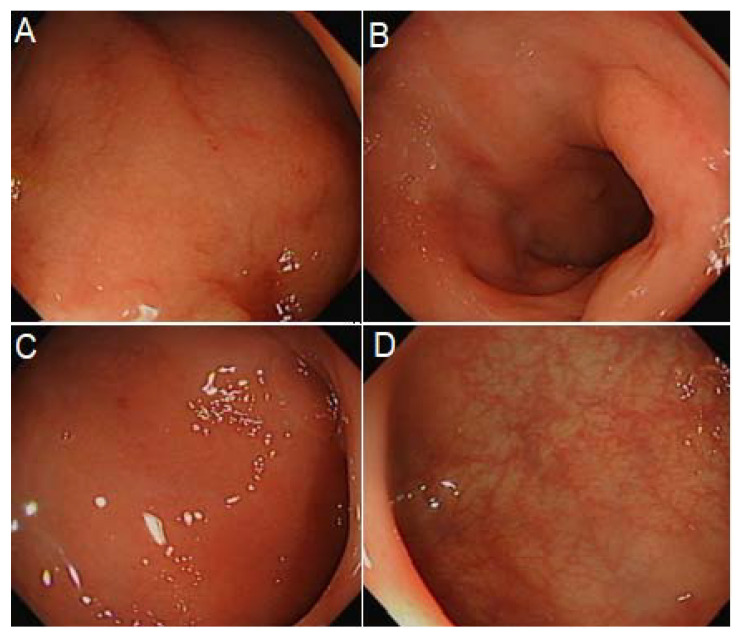
From rectum to splenic flexure (**A**–**D**): findings of proctocolitis, however, improved compared with previous sigmoidoscopy.

After completing the course of ganciclovir, there was an improvement in symptoms and performance status. She had a repeat sigmoidoscopy which showed improvement in colitis (Figure 3), with subsequent biopsies confirming the resolution of the viral complication.

**Figure 4:**
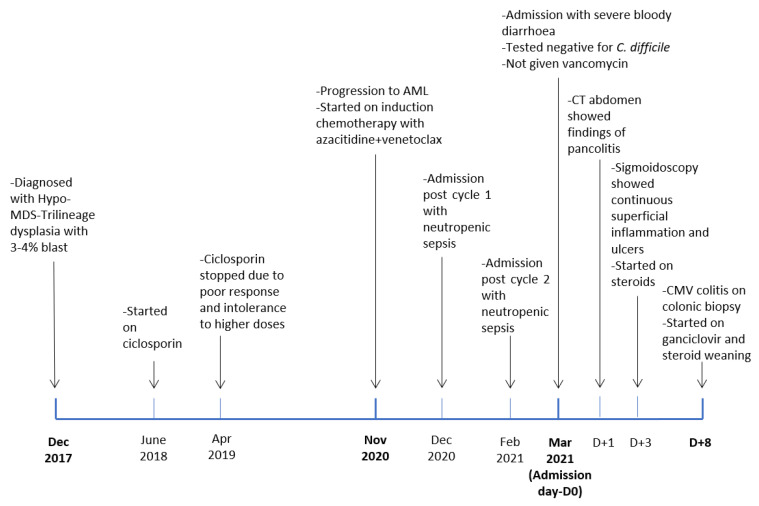
Timeline of events.

Given that complication, she was not considered fit enough for further chemotherapy and eventually for an allo-HSCT. She is now having regular follow-ups with the possibility of chemotherapy in case of relapse. A timeline of events is shown in Figure 4.

## 3. Discussion and Conclusions

Symptoms of CMV colitis are rather non-specific, ranging from diarrhoea, rectal bleeding, fever, abdominal pain, and weight loss, to colonic perforation [18]. Additionally, the presence of raised faecal calprotectin warrants the exclusion of inflammatory bowel disorders. The presence of mucosal toxicity from chemotherapy and either the deficiency of vitamin D or dysbiosis can predispose patients to a more severe phenotype of gastrointestinal toxicities from chemotherapy [19].

Diagnosis involves endoscopy which enables visual assessment and more importantly provides the opportunity to take a biopsy for histopathological examination to ensure a differential diagnosis between IBD and viral infections. The colonoscopic findings of CMV colitis are variable, and usually, diagnosis is confirmed using histological tests such as H&E stain which can show the typical viral inclusions associated with CMV colitis. The gold standard, however, involves the use of CMV-specific immunohistochemistry, labelling CMV antigen in infected cells [20].

The first-line treatment in haematology patients in non-transplant settings presenting with symptomatic CMV disease is intravenous ganciclovir. Ganciclovir should be administered at the standard dosage of 5 mg/kg for 7 to 14 days. In some instances, ganciclovir can cause neutropenia, predisposing the patient to a secondary infection, in which case intravenous foscarnet can be used [2].

**Figure 5:**
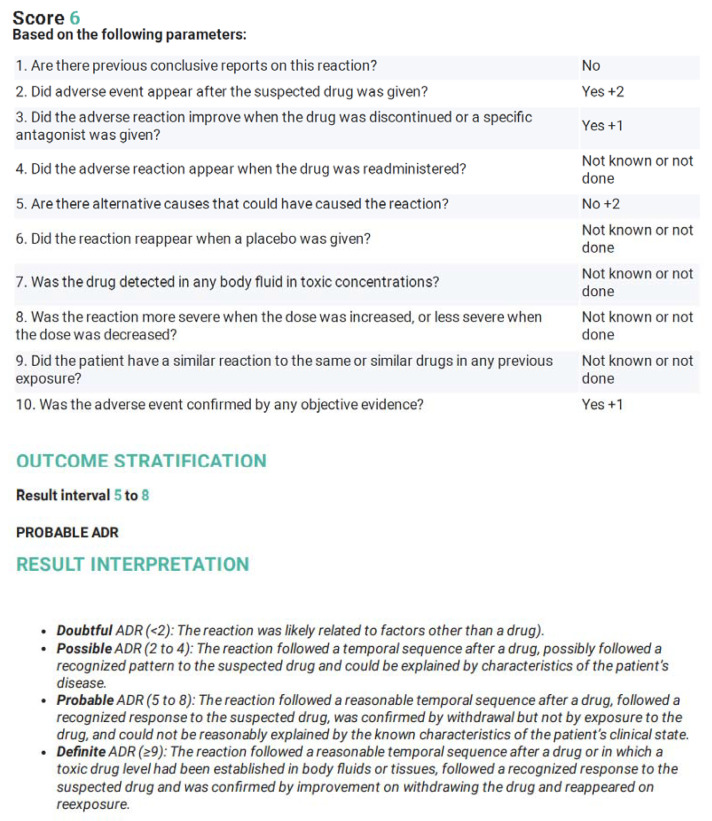
Naranjo Adverse Drug Reaction Probability Scale (Taken and adapted from www.evidencio.com/models/show/661, accessed on 8 March 2022).

A score of 6 was obtained when we used the Naranjo Adverse Drug Reaction Probability Scale (Figure 5), to prove the causative relationship of the chemotherapy drugs to CMV colitis. Based on this scale, a score of 5–8 is indicative of a probable adverse drug reaction. In our patient, there was a clear, temporal association between chemotherapy administration and the development of symptoms.

As mentioned above, there is already a previous case report that highlights the recurrence of CMV colitis following azacitidine administration. Our case adds more weight to this association and serves as a reminder for haematologists to consider CMV disease as a possible complication of azacitidine administration, in light of the fact that the combination of azacytidine and venetoclax is currently considered the standard of care for frail patients affected with AML.

## Figures and Tables

**Table 1:** Cases of CMV colitis arising post-chemotherapy.

Author	Cancer Type (Solid vs. Haem)	CMV Colitis (Yes/No)	CMV Viraemia (Yes/No)	Previous Transplant (Yes/No)	Chemotherapy Regimen during the CMV Episode	Numbers of Cycles of Chemo to CMV Reactivation	CMV-Death
Teraishi F [6]	Solid	Yes	Not available	No	5-FU, Leucovorin, Irinotecan	1	not available
Chuang T M [7]	Haem	Yes	No	No	Dasatinib	Not available	No
Bossa F [8]	Solid	Yes	Not available	No	Ipilimumab	Not available	Not available
Van Den Brande J [9]	Solid	Yes	Not available	No	5-FU, docetaxel, Cisplatin	1	No
Hayashi H [10]	Solid	Yes	Not available	No	Uracil-tegafur	Not available	No
An J [11]	Haem	Yes	Not available	No	Cytarabine, Mitoxantrone	1	No
Polprasert C [12]	Haem	Yes	Not available	No	Rituximab-Cyclophosphamide-Vincristine-Prednisolone	8	No
Nomura K [13]	Haem	Yes	Not available	No	Rituximab, Cyclophosphamide, Adriamycin, Vincristine, Prednisolone	6	No
Matthes T [14]	Solid	Yes	Yes	No	Cisplatin, Etoposide	1	No
Case Jr R [15]	Solid	Yes	Yes	No	Capecitabine	3	No
Khan R [4]	Haem	Yes	Not available	No	Azacitidine	5	No
Current case	Haem	Yes	no	N	Azacitidine + venetoclax	2	No

**Table 2:** Complete blood counts.

CBC Component	Result	Reference Range
White cell count	7.79 × 10^9/L	4.00–11.00 × 10^9/L
Neutrophils	5.74 × 10^9/L	2.2–6.3 × 10^9/L
Lymphocytes	0.75 × 10^9/L	1.3–4.0 × 10^9/L
Monocytes	0.58 × 10^9/L	0.2–1.0 × 10^9/L
Basophils	0.05 × 10^9/L	0–0.1 × 10^9/L
Eosinophils	0.68 × 10^9/L	0–0.4 × 10^9/L
Haemoglobin	101 g/L	115–155 g/L
Platelet	404 × 10^9/L	150–450 × 10^9/L

**Table 3:** Stool test panel.

**Faecal Virology Test**	
Faecal adenovirus DNA	Negative
Astrovirus	Negative
Norovirus	Negative
Sapovirus	Negative
Rotavirus	Negative
**Faecal Bacteriology Test**	
Salmonella	Not isolated
Shigella	Not isolated
Campylobacter	Not isolated
*E. coli* 0157	Not isolated
Clostridium difficile toxin	Negative
**Faecal Parasitology Test**	
Cryptosporidium ZN stain	Not seen
**Marker for Inflammatory Bowel Disease**	
Faecal Calprotectin level	2260 [<50 ug/g]

## Data Availability

Not applicable.
